# Differential Centrifugation to Enrich Bacterial Ribonucleoprotein Bodies (BR bodies) from *Caulobacter crescentus*

**DOI:** 10.1016/j.xpro.2020.100205

**Published:** 2020-12-09

**Authors:** Nisansala S. Muthunayake, Nadra Al-Husini, Jared M. Schrader

**Affiliations:** 1 Wayne State University, Department of Biological Sciences, Detroit, MI 48202, USA

**Keywords:** Subject Areas: Cell Biology, Molecular Biology, Protein Biochemistry

## Abstract

Bacterial RNP bodies (BR bodies) contain the mRNA decay machinery, but the collection of associated RNAs and proteins are poorly defined. Here, we present a protocol for the rapid differential centrifugation-based enrichment of BR bodies from *Caulobacter crescentus* cells. As native BR bodies are highly labile and dissociate by degrading internal mRNAs, an active site mutant of RNase E, which blocks dissolution of BR bodies, allows BR-body stabilization during enrichment.

For complete details on the use and execution of this protocol, please refer to Al-Husini et al. (2020).

## Highlights

• This protocol describes rapid enrichment of BR bodies from *Caulobacter* cells
• An RNase E active site mutation stabilizes the BR bodies during enrichment
• BR-body-enriched pellet can be used to extract RNA and proteins

## Graphical Abstract

**Figure:**
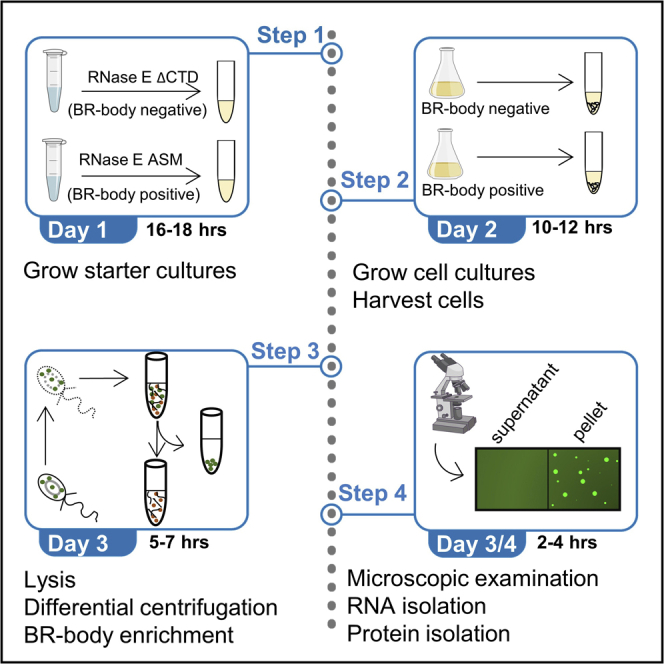

## Before You Begin

### Experimental Preparations

**Timing: 0.5–4 h**

1. If needed, prepare buffers and cell growth media. Make sure that there is enough of all solutions that are needed before cell growth.
2. Streak the strains of interest (JS299 and JS221 BR-body deficient negative control) on appropriate selection plates (peptone-yeast extract (PYE), 5 μg/mL kanamycin, 0.5 μg/mL gentamicin, 0.2% xylose, 1.5% agar) and incubate overnight (~ 16 h) at 28°C.

**CRITICAL:** RNA can easily undergo degradation due to RNase contaminations. Therefore, to avoid RNase contaminations ensure all reagents, buffers and equipment are RNase-free. Diethyl pyrocarbonate (DEPC)-treated water can be used to make buffers.

***Note:*** Importantly, as native BR bodies are highly labile and not detectible in a cell lysate, this protocol utilizes a strain with an RNase E active site mutation (ASM, JS299 *NA1000 vanA::RNE (ASM) YFP RNE::pXRNEssrAC GentR KanR*) to stabilize the bodies. As a negative control, a strain harboring an RNase E C-terminal domain truncation (JS221 *NA1000 vanA::RNE(NTD)-YFP RNE::pXRNEssrAC GentR KanR*) that cannot assemble BR bodies can be utilized (Al-Husini et al., 2020).

***Note:*** All *Caulobacter crescentus* strains used in this study were derived from the wild-type strain NA1000 (Evinger and Agabian, 1977) and were grown at 28°C in PYE medium. The optimal incubation temperature and growth medium will differ based on the type of bacteria.

## Key Resources Table

**Table:** 

REAGENT or RESOURCE	SOURCE	IDENTIFIER
**Bacterial Strains**		

*Caulobacter crescentus* NA1000	Lucy Shapiro, Stanford University School of Medicine	N/A
JS 299 strain *NA1000 vanA::RNE(ASM)YFP RNE::pXRNEssrAC GentR KanR*	Schrader lab, Wayne State University	N/A
JS 221 strain *NA1000 vanA::RNE(NTD)-YFP RNE::pXRNEssrAC GentR KanR*	Schrader lab, Wayne State University	N/A

**Chemicals, Peptides, and Recombinant Proteins**		

Vanillic acid	Fluka	94770-50G
Xylose	Sigma-Aldrich	X1500-500G
Gentamycin	Sigma-Aldrich	G1264-5G
Kanamycin	Sigma-Aldrich	K1377-5G
Sodium chloride	Sigma-Aldrich	BP358-1 -1KG
Tris-HCl	Ambion	AM9851 -500ML
β-Mercaptoethanol	Amresco	M131-100ML
EDTA-free protease inhibitor	Roche	11873580001
RNase-free DNase I	Roche	04 716 728 001
Superase inhibitor	Invitrogen	AM2694
TRizol reagent	Ambion	15596018
Chloroform	Sigma-Aldrich	496189-1L
Isopropanol	Sigma-Aldrich	34863-4L
Peptone	BD Biosciences	211677-500G
Agar	BD Biosciences	214010-454G
Yeast extract	Fluka	92144-500G
Calcium chloride	Sigma-Aldrich	746495-500G
Magnesium sulfate	Sigma-Aldrich	M7506-500G
Ferrous sulfate	Sigma-Aldrich	F0518-1L
Glucose	Sigma-Aldrich	G8270-1KG
Sodium dodecyl sulfate	Sigma-Aldrich	436143-100G

**Critical Commercial Assays**		

Qubit RNA HS Assay Kit	Thermo Fisher	Q32852
Bioanalyzer RNA 6000 Nano Kit	Agilent	N/A

**Software and Algorithms**		

ImageJ	(Rueden et al., 2017)	https://imagej.nih.gov/

**Other**		

Standard shaker incubator	N/A	N/A
Nanodrop spectrophotometer	Thermo Fisher	N/A
JA-20 fixed-angle aluminum rotor	Beckman Coulter	N/A
Mixer mill MM 400	Roche	N/A
Grinding jars 10 mL	Roche	N/A
Grinding balls 12 mm	Roche	N/A
Refrigerated centrifuge	Thermo Fisher	N/A
Microscope	Nikon	N/A
Bioanalyzer	Agilent	N/A
Qubit 4 Fluorometer	Thermo Fisher	N/A
Microscope	Nikon	Ni-E
CCD camera	CoolSnap	Myo
YFP filter cube	Chroma	96363
Microscope cover glass	Fisher brand	12-545-M
Microscope slide/white epoxy ink frosted end one side	Thermo Fisher	n/a
Immersion oil for microscopy	Nikon	Type A
Standard microcentrifuge	n/a	n/a
Standard Thermomixer	n/a	N/A

## Materials and Equipment

**Table:** PYE Medium

Chemical	Final concentration	Stock Concentration	Add to 1,000 mL
Bactopeptone	2 g/L	N/A	2 g
Yeast Extract	1 mg/L	N/A	1 g
MgSO_4_	1 mM	0.5 M	2 mL
CaCl_2_	0.5 mM	0.1 M	5 mL
ddH_2_O			993 mL
**Total**			**1,000 mL**

***Note:*** Store the autoclaved PYE medium at 25°C.

**Table:** M2G Medium

Chemical	Final concentration	Stock Concentration	Add to 100 mL
MgSO_4_	0.5 mM	0.5 M	100 μL
FeSO_4_	0.5 mM	0.5 M	100 μL
CaCl_2_	0.5 mM	0.1 M	500 μL
Glucose	0.2%	20%	1 mL
ddH_2_O			98.3 mL
**Total**			**100 mL**

***Note:*** Store the autoclaved M2G medium at 25°C.

**Table:** Lysis Buffer

Reagent	Final concentration	Stock Concentration	Add to 25 mL
NaCl	35 mM	1 M	875 μL
Tris-HCl-pH 7.4	20 mM	1 M	500 μL
β-Mercaptoethanol	1 mM	13.5 M	2 μL
Superase inhibitor	1 U/mL	1,000 U/mL	25 μL
RNase-free DNase I	10 U/mL	10,000 U/mL	25 μL
EDTA-free protease inhibitor	N/A	N/A	1 tablet
ddH_2_O			23.5 mL
**Total**			**25 mL**

***Note:*** Store the lysis buffer in 4°C.

**CRITICAL:** β-Mercaptoethanol is volatile and toxic so ensure that you open concentrated β-mercaptoethanol bottles in a chemical fume hood.

***Alternatives:*** β-ME can be replaced by the less toxic dithiothreitol (DTT) alternative (Mommaerts et al., 2015).

## Step-By-Step Method Details

### Prepare Initial Cultures: Day 1

**Timing: 16–18 h**

This step describes how to culture *Caulobacter* starting from freezer stocks.

1. Use a scrape of a freezer stock to inoculate each bacterial strain in 5 mL PYE medium with 0.2% xylose, 0.5 μg/mL gentamycin, and 5 μg/mL kanamycin in a 10 mL test tube.
2. For each strain, make three 1: 20 serial dilutions (5 mL each in 15 mL test tubes) and incubate the cultures in a shaker incubator at 28 ˚C while shaking at 250 rpm overnight (~16 h).

***Note:*** All cell growth incubations are performed at 28 ˚C in a shaker/incubator at 250 rpm in PYE growth medium. Make sure to mix the culture tube well to have a homogeneous cell suspension before preparing the dilutions. The purpose of serial dilution is to get a bacteria culture with appropriate cell density and in log-phase of growth to inoculate the large culture. It prevents giving high dense overnight cultures.

***Note:*** In working with *Caulobacter crescentus* depletion strains, it can be useful to validate the depletion phenotype by growing the cells in PYE supplemented with 0.2% xylose and without. The cells should exhibit growth only in the presence of xylose. If growth is not xylose dependent, this indicates that the culture has a suppressor mutation and one needs to re-streak the culture to identify a colony which requires xylose for growth.

### Grow and Harvest Cell Cultures: Day 2

**Timing: 10–12 h**

This step describes how to grow and harvest *Caulobacter* cells.

**Figure 1:**
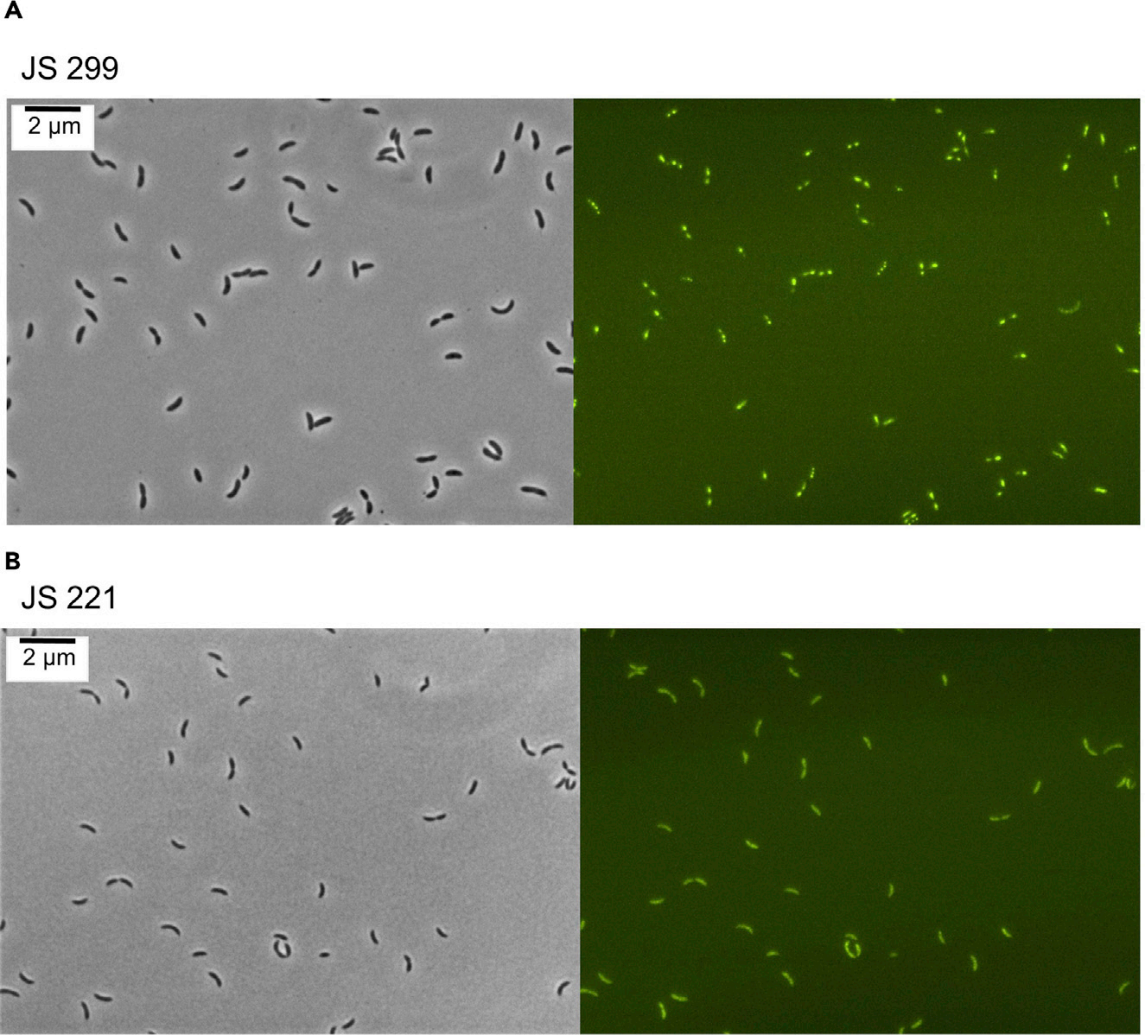
BR-Body Verification before Cell Harvesting (Step 7) (A) The formation of RNase E-YFP foci is observed in the ASM strain (JS 299). (B) Diffuse localization of the RNase E-YFP CTD deletion mutant (JS221), which cannot assemble BR bodies.
8. To harvest, transfer cells to a 50 mL conical tube chilled on ice, and pellet cells at 11,000 × *g* for 5 min at 4°C. Remove the supernatant and resuspend the cell pellet in 2.5 mL of ice-cold lysis buffer.
9. Slowly drip the cell pellet/lysis buffer suspension into a 50 mL conical tube half-full of liquid nitrogen. Poke holes in a 50 mL conical lid using a hot needle and pour out remaining liquid nitrogen in the tube. Once liquid nitrogen has completely evaporated, cap with a new lid and store the cell pellet in a −80°C freezer.

***Note:*** Cultures should be grown for 8 h until they reach mid-exponential phase of growth (OD 0.4–0.6). It takes approximately 8 h for *Caulobacter* to fully replace the wild-type RNase E expressed from the xylose promoter with the RNase E variant expressed from the vanillate promoter. The optimal timescale for replacement was determined by western blot (Al-Husini et al., 2018) and changes to this timing may alter the amount of active RNase E present in the cell.

***Note:*** It is important to examine and confirm the formation of RNase E–YFP foci by imaging the cells after few hours of induction. In this specific protocol, after 4 h and 8 h of incubations 1 μL of cells were spotted onto a M2G 1.5% agarose pad, air dried, and imaged with immersion oil on a coverslip (Figure 1). Images were taken in phase-contrast and fluorescence (YFP) using a filter cube.

***Note:*** Optical density should be measured at 600 nm in a cuvette with a 1 cm path length.

3. Wash the overnight culture 3× with PYE growth media and resuspend the washed cells in fresh 5 mL of PYE growth media.
4. Take a 1 mL aliquot of the culture and measure the optical density (OD_600 nm_).
5. Dilute the culture in 30 mL of PYE medium supplied with vanillate (500 μM), gentamycin (0.5 μg/mL) and kanamycin (5 μg/mL) to an OD_600 nm_ of 0.05 in a 125 mL Erlenmeyer flask. Mix well and measure the starting OD_600 nm_ of the culture by taking a 1 mL aliquot.
6. Incubate cells at 28 ˚C and 250 rpm and measure the OD_600 nm_ every 2 h until it reaches ~0.4–0.6.
7. Confirm presence of BR bodies by imaging a 1 μL aliquot of both JS299 and JS221 strains on M2G 1.5% agarose pads (Skinner et al., 2013) before harvesting the cells (Figure 1).

**CRITICAL:** Use cryogenic gloves and a face shield when working with liquid nitrogen.

### Cell Lysis and BR-Body Enrichment: Day 3

**Timing: 5–7 h**

10. Lyse cells in a mixer miller
  **Timing: 1–2 h**
  This step describes how to cryogenically lyse bacteria cells using a mixer miller.
  a) Chill assembled jar (10 mL) and grinding ball (12 mm) in liquid nitrogen.
  b) Open the pre-chilled jar and add the frozen cell pellet/lysis buffer suspension.
  c) Mill at 15 Hz for 3 min. Re-chill the sealed jar in liquid nitrogen until it stops bubbling between each run. Repeat for five total cycles of milling.
  d) Briefly pre-chill a scoopula and 1.5 mL microcentrifuge tube in liquid nitrogen. Open the jar, and take a small scoop of the frozen pulverized cells using a pre-chilled scoopula and transfer it to a pre-chilled 1.5 mL microcentrifuge tube labeled “whole-cell lysate” and store at −80°C.
  e) To thaw the remaining lysate, place each half, chamber side up, in a shallow pool of warm 30°C water. Be careful that the water level is low enough that it will not leak into the jars.

**CRITICAL:** Lysing for too long and too continuously in a small volume may cause heating of the sample; this may result in degradation of the RNA. Therefore, samples should be kept frozen during lysis, and mixing should be done in short on-off cycles with care to ensure the jar is properly chilled. Once thawed, the BR bodies are labile so care should be taken to work quickly.

***Note:*** Use cryogenic gloves and a face shield when working with liquid nitrogen.

***Note:*** Since BR bodies are highly labile upon cell lysis, cryogenic mixer milling allows a significant reduction in the time the lysate is thawed, allowing higher yields of BR bodies than methods performed on liquid-samples such as French press or sonication.

**Figure 2:**
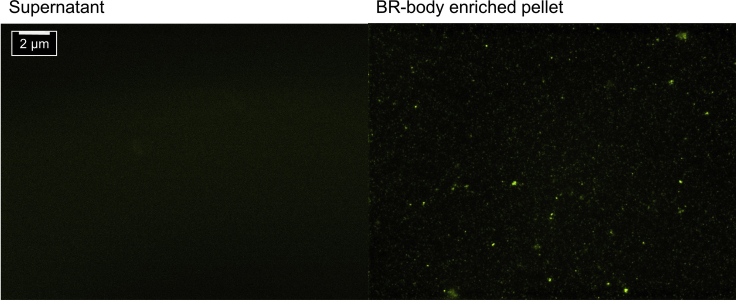
BR-Body Enrichment Fluorescence microscopy images of ASM-YFP were performed with pellets resuspended in the same volume as supernatant for comparison (step 11e).

***Note:*** The BR-body enrichment procedure with differential centrifugation is adapted from (Wheeler et al., 2017, Khong et al., 2017, Khong et al., 2018).

***Note:*** A small amount (~2–5 μL) of the sample from each centrifugation step should be saved to monitor the enrichment procedure progress by fluorescence microscopy. The liquid sample (~ 5 μL) was spotted onto a microscope slide and covered with a coverslip and put an oil droplet prior to imaging with a 100× objective. Images were taken in both phase-contrast and fluorescence (YFP) imaging. All images were processed using ImageJ (Figure 2).

11. Differential Centrifugation steps
  **Timing: 30 min to 1 h**
  This step describes the differential centrifugation steps involved in BR-body enrichment.
  a) Move the thawed lysate into a 1.5 mL Eppendorf tube and spin at 2,000 × *g* for 5 min to remove cell membranes, intact cells, and large cell debris.
  b) Carefully transfer the supernatant into a fresh 1.5 mL tube and spin at 10,000 × *g* for 10 min.
  c) Resuspend the resulting BR-body pellet by gently pipetting up and down in 1.2 mL of lysis buffer and spin at 20,000 × *g* for 10 min.
  d) For RNA isolation, resuspend the BR-body-enriched pellet in 200 μL of lysis buffer, and proceed to step 12. For protein isolation, resuspend the final pellet in 200 μL of lysis buffer containing 1% SDS, and proceed to step 13.
  e) Confirm presence of BR bodies by imaging a 5 μL aliquot of the final pellet (Figure 2).

JS221 can be used as a negative control in which no BR bodies will be detected. Focusing on the BR bodies can be difficult as they can photobleach rapidly. Be sure to change field of view frequently while attempting to focus the microscope. Once focused, a microscope with a digital piezo Z-axis can be much more easily kept in focus for each subsequent sample.

12. RNA isolation
  **Timing: 2–4 h**
  This step describes how to isolate RNA from the BR-body enriched pellet.
  a) Preheat 1 mL of Trizol for each biological sample at 65°C in the heat block.
  b) Add 1 mL of 65°C Trizol to the samples, mix well by vortexing and incubate at 65°C for 10 min.
  c) Then add 200 μL of chloroform, mix well by vortexing and incubate for 5 min at room temperature (~25°C).
  d) Spin the samples at max speed (20,000 × *g*) in a microcentrifuge for 10 min at room temperature (~25°C).
  e) Remove the aqueous layer and precipitate with 700 μL of isopropanol at −20°C for 1 h.
  f) Spin the samples at 20,000 × *g* for 1 h at 4°C.
  g) Decant off the supernatant carefully and wash the pellet (slightly white color) with 800 μL of 80% ethanol by shaking the tube several times.
  h) Spin the samples at 20,000 × *g* for 1 min at 4°C. Repeat steps (g) and (h) three times.
  i) Discard 80% ethanol and air dry the pellet and resuspend in ~50 μL 10 mM Tris pH 7.0.
  j) Assess RNA quality using Bioanalyzer and RNA 6000 Nano Kit, following manufacturer’s instructions.

***Note:*** For RNA isolation step it is recommended to use RNase-free, siliconized polypropylene microcentrifuge tubes, which provides extremely low surface adhesion and enables maximum sample recovery.

***Note:*** It is possible to use Bio analyzer to determine RNA concentration. However, Qubit is slightly better (accurate) than Bioanalyzer and is recommended if the RNA is going to be prepared for RNA sequencing.

13. Protein isolation (extract and quantify protein from both whole-cell lysate and BR-body-enriched fractions)
  **Timing: 2–4 h**
  This step describes how to isolate proteins from the BR-body enriched pellet.
  a) Collect BR-body-enriched and whole-cell lysate fractions from each bacteria strain as explained above.
  b) Resuspend the final pellet in 200 μL of lysis buffer containing 1% SDS.
  c) Quantify the concentration of proteins in each fraction using Bradford assay.

***Note:*** Extracted samples can be used to perform mass spectroscopic analysis to identify the protein composition of BR-body-enriched fraction.

## Expected Outcomes

**Figure 3:**
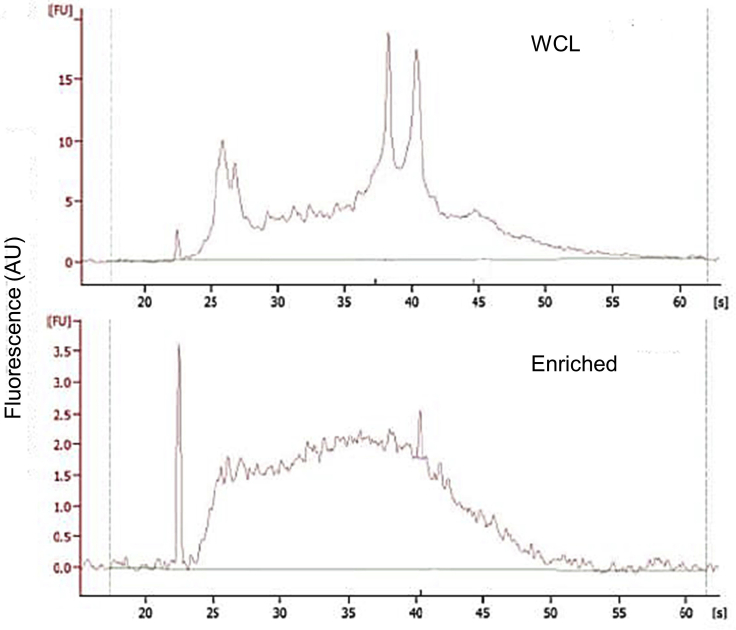
Bioanalyzer Traces of Total RNA Extracted from the WCL Fraction (Top) or from the Enriched Samples Generated from Differential Centrifugation (Bottom) (Step 12 j) Samples were collected from the ASM (JS299) strain. Note that the Y-axis scales are different between the two samples. Figure from Al-Husini et al. (2020).

**Figure 4:**
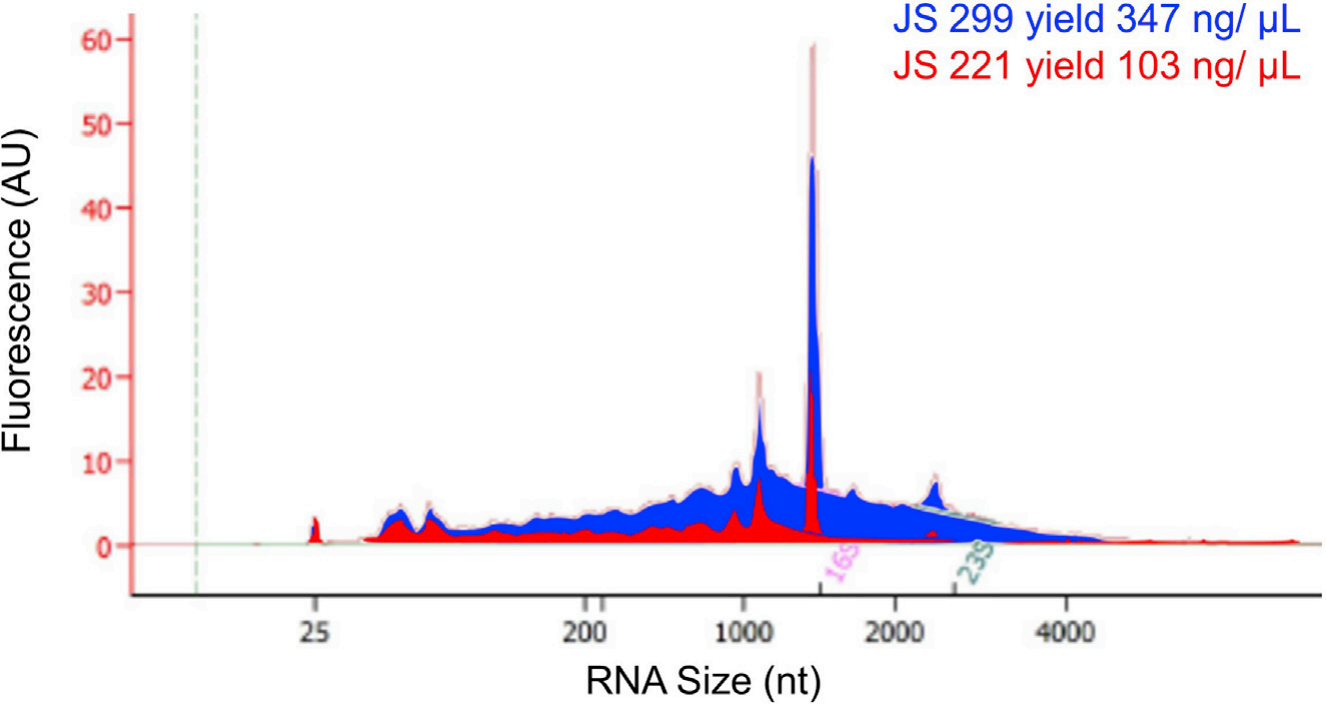
RNA from Final Pelleted Samples Collected from a Mock Sample Generated from the NTD Strain (JS221, Red) and BR Bodies Generated from the ASM Mutant (JS299, Blue) (Step 12 j) Note the NTD strain yielded less RNA and lacked the longer RNA species present in the ASM sample. Figure from Al-Husini et al. (2020).

Importantly, high-yield RNA isolation is an indication of successful enrichment of BR bodies. The whole-cell lysate is rich in rRNA and tRNA, while the BR-body enriched samples are highly depleted of these RNAs (Figure 3). In typical preps, the CTD truncation mutant (JS221) yields 3.5 fold lower RNA levels compared to the ASM (JS299) (Figure 4, Table 1).

**Table 1:** RNA Yield of the BR-Body-Enriched Fraction from Each Bacteria Strain (Step 12 j)

Strain	RNA Yield of the BR-Body-Enriched Fraction
JS 299	347 ng/μL
JS 221	103 ng/μL

Data are from Al-Husini et al. (2020).

## Limitations

Since this protocol takes significant time to complete, it is possible that non-BR-body molecules may associate during the enrichment protocol. It is also possible that some BR-body associated molecules may dissociate during the enrichment protocol.

Since this protocol does not specifically purify BR bodies from other macromolecular complexes that could pellet at the same speed, it is possible that some macromolecules identified are not associated with BR bodies. Therefore, it is crucial to use the JS221 strain as a negative control to subtract these types of macromolecules.

## Troubleshooting

### Problem

Poor cell lysis (step 10)

### Potential Solution

**Figure 5:**
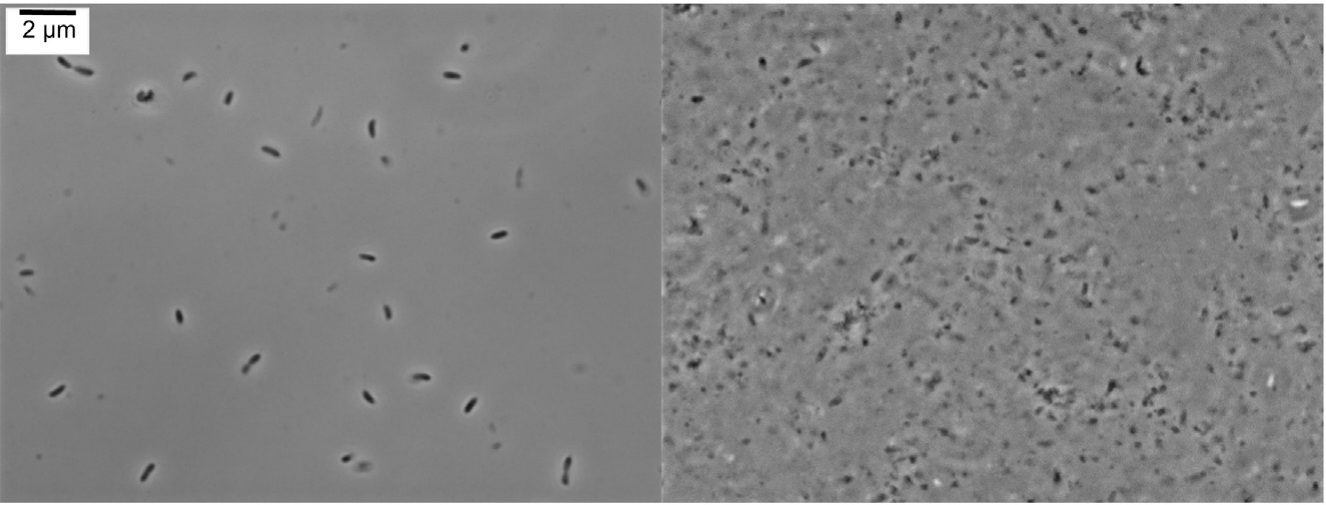
Cell Lysis Using Cryo-Mixer Milling (Step 10) Unsuccessful cell lysis (left) and successful cell lysis (right).

**Figure 6:**
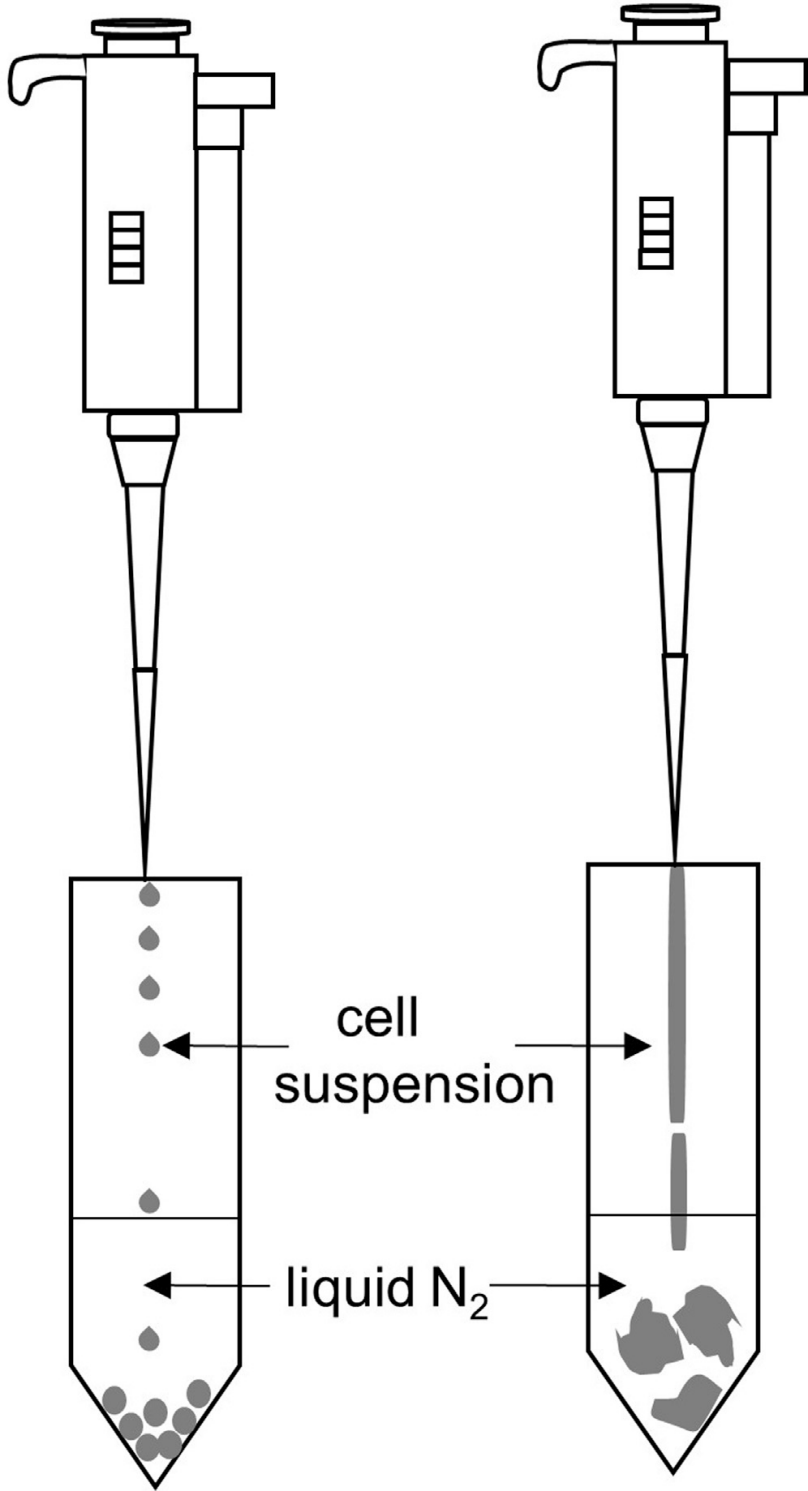
Flash Freezing (Step 9) Drip cell lysate slowly and drop wise to liquid nitrogen in order to get uniform small balls of frozen cell lysate. Successful (left) and unsuccessful flash freezing (right).

The most common problem encountered in this procedure is incomplete cell lysis (Figure 5). The presence of a small number of large chunks of frozen cell lysate generated upon initial harvesting may reduce the extent of cell lysis. Therefore, during the flash freezing step (step 9) make sure to drip cell lysate slowly and drop wise to liquid nitrogen in order to get uniform small balls of frozen cell lysate to ensure good lysis (should look like dipin’ dots ice cream, https://en.wikipedia.org/wiki/Dippin%27_Dots) (Figure 6). It is also very important before milling and between the grinding cycles the grinding jars filled with sample and ball have been cooled in liquid nitrogen. Make sure to use the appropriately sized grinding jars and balls for the mixer miller lysis. For the complete disruption of ~ 4- 10 mL frozen bacteria cell pellet it is recommended to use 10 mL stainless steel grinding jars and 12 mm ball in Cryo Mill.

### Problem

RNase contamination and degradation of RNA.

### Potential Solution

Throughout the protocol there are many steps in which exogenous RNase contamination may destroy the final RNA integrity. Even trace quantities of RNase can lead to degradation during RNA purification protocols. The major sources of RNase contamination in a typical laboratory include aqueous solutions, reagents used in experiments, aerosolized RNases, or inadvertent contact with dust or skin. Most importantly common sense laboratory practices such as wearing gloves during an experiment and change them often, especially after contact with skin, hair, or other potentially RNase-contaminated surfaces, maintaining a separate area for pipettes for RNA work and using RNase-free reagents and microcentrifuge tubes are very helpful to prevent RNase contaminations (Nilsen, 2013). It is recommended to use RNaseZap RNase decontamination solution or similar products to decontaminate glass and plastic surfaces.

### Problem

Overdrying the RNA pellet (step 12i).

### Potential Solution

This is a common issue associated with Trizol extracted RNA (Rio et al., 2010). Overdrying of the RNA pellet is a possible reason for the poor solubility of RNA after precipitation. Therefore, Trizol obtained RNA pellet should not dry under speedvac since it can easily lead to overdrying of the sample. It is recommended to dry the RNA pellet in room temperature (~25°C) about 5 min. Additionally, the use of excess centrifugation speeds during the precipitation also makes it hard to dissolve the final RNA pellet. Therefore, it is critical to use the recommended centrifugation speeds or utilize a coprecipitant like glycoblue that can allow you to track the pellet.

### Problem

Insufficient washing of the BR-body enriched pellet (step 11c).

**Figure 7:**
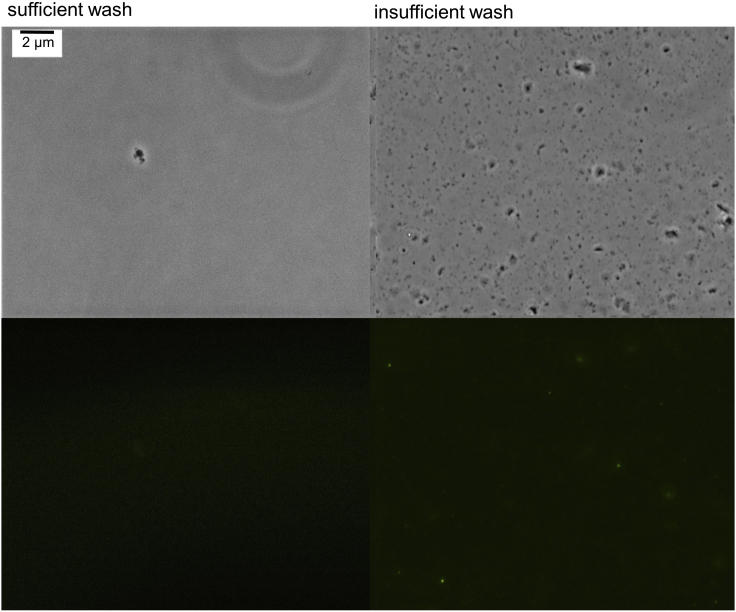
Final Supernatant Insufficient wash (right) and sufficient wash (left).

If we use too little wash buffer or do not decant off enough of the wash buffer after pelleting then samples can suffer lower levels of BR-body enrichment. Insufficient washing can be visualized by the presence of non-fluorescent phase-dark particles in the wash supernatant after pelleting (Figure 7).

### Potential Solution

Make sure to use the recommended number of cells and wash volumes. Be careful when removing supernatant of BR-body enriched pellets as not to disturb pellet.

### Problem

Difficulties in imaging live bacteria cells (step 7).

### Potential Solution

Use agarose pads to mount bacteria cells on the microscopic slide.

**Figure 8:**
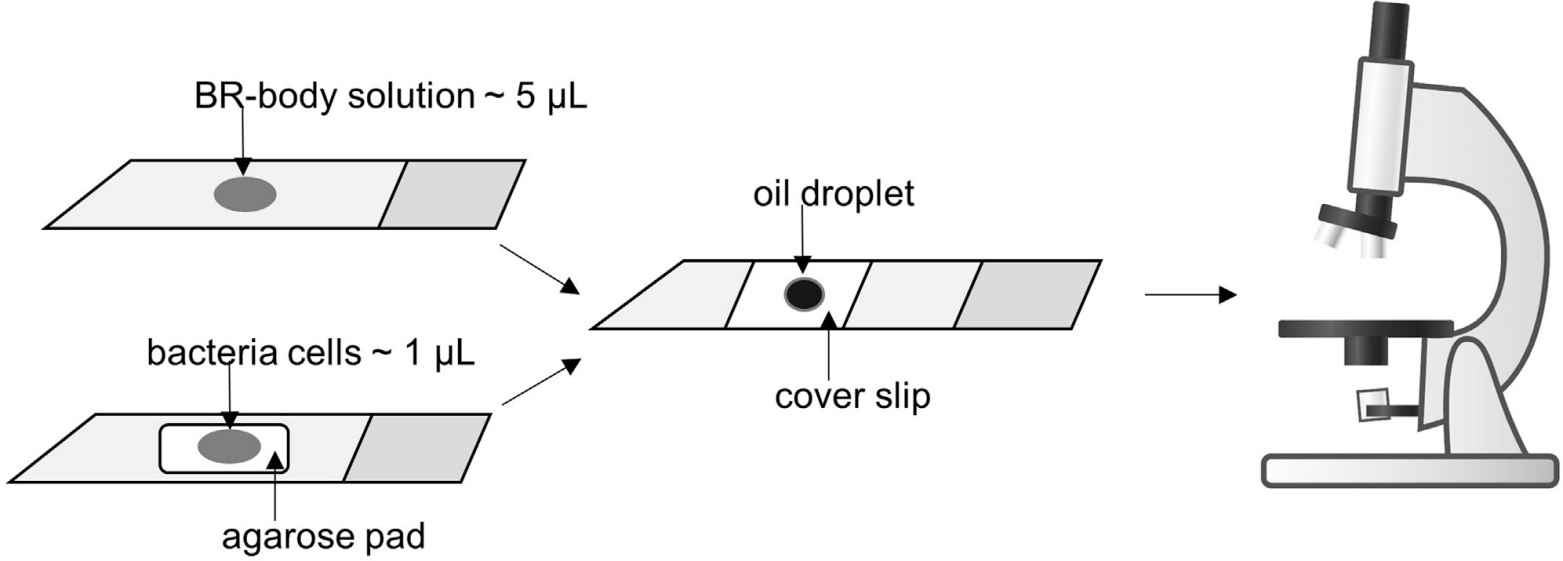
Preparation of Microscopic Slide to Image BR-Body Solution (Top) versus Live Bacteria Cells (Bottom) Microscope image was generated with chemix.org.

It is very difficult to image live bacteria cells since they are non-adherent, and are often motile. This problem can be overcome by using agarose pads to immobilize the cells during the imaging process (step 7). Place a M2G 1.5% agarose pad on the microscopic slide and spot ~1 μL of cells. Air dry and image with immersion oil on a coverslip (Figure 8). During imaging, there might be areas near the edge of the agarose pad where the bacteria will be moving. They are more likely to be static toward the center of the pad. However, to image BR bodies (step 11e) we can directly use a liquid mount (~5 μL) of the BR-body solution to prepare the imaging chamber slide (Figure 8).

## Resource Availability

### Lead Contact

Further information and requests for resources and reagents should be directed to and will be fulfilled by the Lead Contact, Jared M. Schrader (Schrader@wayne.edu).

### Materials Availability

Strains JS299 and JS221 are available from the Schrader lab upon request.

### Data and Code Availability

This protocol does not include any code or datasets.
